# Children and young people’s mental health in the English-speaking Caribbean: a scoping review and evidence map

**DOI:** 10.1186/s13034-021-00435-w

**Published:** 2021-12-30

**Authors:** Shaun Liverpool, Brent Pereira, Malika Pollard, Jamal Prescod, Catherine Trotman

**Affiliations:** 1 Faculty of Health, Social Care and Medicine, Edge Hill University, Ormskirk, UK; 2 Evidence Based Practice Unit, Anna Freud National Centre for Children and Families, University College London, London, UK; 3 Department of Counselor Education, The Chicago School of Professional Psychology, Chicago, USA; 4 Faculty of Social Sciences, University of the West Indies, Cave Hill, Wanstead, Barbados

**Keywords:** Child, Adolescent, Young adult, Mental health, Caribbean region, Minority groups

## Abstract

Internationally, there is a wealth of research suggesting that many children and young people experience mental health problems. However, the evidence from low- and middle-income countries and developing nations is generally limited. This scoping review aimed to add to the body of evidence by providing an overview of the available research literature on children and young people’s (CYP’s) mental health in the English-speaking Caribbean region. Seven key online academic databases and grey literature sources were searched until January 2021. Records were screened against predefined criteria and suitable articles retrieved. Relevant information was then charted and summarized. All stages of the review were informed by expert consultations. Ninety-six articles from 7901 records met the inclusion criteria. Most of the studies were conducted in Jamaica, Trinidad and Tobago and Barbados while fewer studies reported findings from St Lucia, The Bahamas and St Kitts and Nevis. Research funding was not frequently reported, and participants were predominantly recruited in education settings. There was a substantial focus on depressive symptoms and behaviour problems. Little or no research was available for younger children (< 12), complex clinical cases or commonly under-represented groups. Four unique interventions were identified of which one intervention showed no significant impact on CYP. Among the commonly used outcome measures only the Jamaican Youth Checklist (teacher-reported), Beck Depression Inventory-II, Brief Screen for Depression, Trinidad and Tobago Youth Survey and Minnesota Multiphasic Personality Inventory had evidence for psychometric validity within this population. We discuss future directions, implications and recommendations for research, practice, policy and training.

**Supplementary Information:**

The online version contains supplementary material available at 10.1186/s13034-021-00435-w.

## Background

Mental health problems have consistently been identified as one of the main causes of overall disease burden and one of the primary drivers of disability worldwide [1–3]. Among children and young people (CYP), 10–20% experience mental health problems [4–6]. The evidence also suggests that 50% of these mental health problems begin by age 14 and 75% by age 24 [4, 7], with anxiety and behavioural problems being among the most common [6, 8]. In addition, suicide, self-harm and other internalising and externalising problems have continued to increase in recent years [4, 8–10]. Previous studies also suggest that CYP’s mental health problems tend to co-occur with other mental or physical health problems and present adverse outcomes in adulthood [9–11]. To fully develop a global mental health perspective, experts advocate for evidence from specific sub-populations [12].

According to the United Nations statistics around 90% of CYP live in low- and middle-income countries [13]. Researchers have identified several risk factors associated with mental health in these countries. Some risk factors include problems in physical health and nutritional status of the child, being raised in institutions, severe physical punishment, academic difficulties, bullying in schools and family dysfunction [5]. Although risk factors may be common across low- and middle-income countries, there is potential that mental health prevalence may vary, owing to the influence of culture on the identification and interpretation of symptoms [14, 15]. As a result, further investigations of smaller regions could broaden our understanding with implications for the development and implementation of tailored services [16, 17]. Other well-documented issues affecting this population have been stigma and the lack of resources which limit the chances for mental health difficulties to be identified and treated [18, 19]. However, with the advancement of technology [20] and its acceptance by children and young people [21], this phenomenon could be revisited with new lens.

Notably, in the last decade, there has been growing concerns about increasing mental health problems in the Caribbean region [22]. A regional study reporting data from nine Caribbean countries on adolescents age 10–18 indicated that 1 in 6 (17%) adolescents saw themselves as sad, angry or irritated [23]. Country specific data also highlighted a 7.4% rate of depression among CYP in Jamaica [24], and 14% in Trinidad and Tobago [25]. Another study conducted in Jamaica, The Bahamas, St Kitts and Nevis and St Vincent and the Grenadines revealed that among the sample of n = 1955 adolescents nearly half of the sample (52.1%) reported mild to severe symptoms of depression and a further 29.1% reported moderate to severe symptoms [26]. A systematic review of the Caribbean literature also confirmed that the incidence of depression as well as the severity of symptoms and outcomes were more common during early and middle adolescence [27]. Another systematic review on general health, conducted almost a decade ago and concentrated on adolescents 10–19 years, identified 40 articles on adolescent mental health [28]. That study used a multisystem framework and explored risk (e.g., age, presence of chronic illness) and protective (e.g., family, religiosity) factors associated with mental health. The previous authors highlighted that the Caribbean evidence is limited and encouraged further research including a broader search of the available literature. It also appeared that the literature may have focused mainly on depression or low mood and limited by age. An updated more comprehensive review of the literature is needed to identify and explore other mental health problems to present a more holistic regional perspective.

At a minimum, the findings of this review can be used to inform future directions for CYP’s mental health research in the Caribbean. For example, it may suggest targeted areas to conduct further evaluations of identified interventions or to collect primary data in areas where a dearth of evidence is observed. Further, any inconsistencies, limitations or lack of resources identified can be used to make or inform future directions and recommendations. Apart from the obvious direct and indirect benefits of carrying out research in the Caribbean, a broader understanding of CYP’s mental health in a Caribbean context could also have implications for international researchers, practitioners and policy makers. First, Caribbean students make up a fair percentage of the international student body at universities around the world [29, 30]. Second, the immigration rates of families from the Caribbean to the Western world have been constant over the years [31]. Third, the extant literature generally acknowledges limitations of ethnic minority groups in research, with valid consequences to generalizability [32]. The current study could help address these areas of interest, by providing a knowledge base on which to draw necessary comparisons.

## Rationale

The importance of identifying, preventing, assessing, and treating/supporting CYP’s mental health have been well established globally. However, the academic discourse around this topic in the Caribbean is either limited or scattered across disciplines (e.g., social work or medicine). Researchers, practitioners, and decision-makers could benefit from an overview of what is available, what is missing and any cultural variations or comparisons within the CYP mental health literature. As such, the findings of this review can be useful in prioritising areas for future directions in the field of CYP’s mental health in Caribbean.

### Aims/objectives

The overarching objective of this scoping study is to provide a preliminary overview of the available research literature on CYP’s mental health and wellbeing in the English-speaking Caribbean region. To achieve this, it was necessary to: (1) search, screen and organise the available research evidence, (2) examine and summarise the extent (size), range (variety), and nature (characteristics) of the research activity, (3) identify and analyse knowledge gaps, (4) record evidence-based interventions and common outcome measures that can be readily implemented or further developed, and (5) conduct consultation exercises to help guide the review process and ensure the findings are fit for purpose.

### Methods

Based on the knowledge synthesis tool [33], a scoping review framework was recommended for exploring and mapping the research on CYP’s mental health in the English-speaking Caribbean region. This scoping review methodology was informed by the framework proposed by Arksey and O’Malley [34] and incorporated updates by Levac et al. [35]. The review process was also guided by the Joanna Briggs Institute recommendations [36] and reported according to the Preferred Reporting Items for Systematic Reviews and Meta-Analyses (PRISMA) extension for scoping reviews [37]. Therefore, steps were taken to identify relevant information, chart the data, and collate and summarise the findings. A protocol was compiled a priori and registered with the Open Science Framework [38].

### Information search and search strategy

The literature search was broad in scope and range to capture the breath of the existing knowledge [39]. Mental health encompassed mental, emotional, psychological, behavioural and social well-being [40] of children (below the age of 14) and young people (15–24) [41] in the English-speaking Caribbean [42]. Key concepts relating to “children OR young people” AND “mental health OR well-being” AND “Caribbean” were selected to inform the search strategy (see Additional file 1). The search strategy was developed and piloted through an iterative process with a research librarian at the University College London Institute of Child Health. Searches were conducted using the CINAHL, Cochrane Library, EMBASE, MEDLINE, PsycINFO, LILACS, and Web of Science databases. Given the importance of the field, the perceived amount of ongoing research in the area, and to increase comprehensiveness, grey literature searches were also conducted in OpenGrey, ResearchGate and the first 10 pages of Google. The reference list of relevant articles and systematic reviews were also scanned for further additional articles related to the review. Citation tracking and reference trolling were used to identify peer-reviewed articles arising from relevant abstracts and theses. Searches were conducted independently and in parallel by two reviewers (SL and JP). All records were transferred to the Mendeley reference manager [43].

### Screening and selection process

After duplicates were removed, a three-step screening process was conducted to identify empirical studies that explored mental health symptoms or diagnoses. First, the reviewers piloted 10 randomly selected articles against the eligibility criteria (Table 1). After discussions and consensus checking, the titles and abstract were independently screened and reported as “include”, “exclude” or “unsure”. Where both reviewers agreed to “include” or were “unsure” those records were retained for full-text screening. Where disagreements occurred, those records were also moved to the full-text screening stage to obtain further information. Third, the full-text versions of the remaining potential records were independently examined for final inclusion. Any disagreements at this stage were resolved through discussions, and when necessary, input from a third member of the team was obtained.

**Table 1:** Eligibility criteria

Criteria	Inclusion	Exclusion
Population	Studies focusing on children and young people (0–24 years); Studies were included if the reported mean or mode age was ≤ 24 years	Studies focusing on adult populations > 24 years; Studies focused on (pregnant) older parents
Concept	Studies exploring mental health symptoms or diagnoses, or mental health difficulties related to a primary physical/somatic condition	Studies with a primary focus on physical health (e.g., cancer and diabetes). Studies were also excluded if they: (a) primarily examined autism spectrum disorders or developmental disorders including intellectual impairment (i.e., mental retardation, severe foetal alcohol syndrome), language/communication disorders (i.e., receptive, expressive disorders) as these were beyond the scope of this study. Substance-use disorders were also excluded as they are often treated outside of generic child and adolescent mental health services
Context	English-speaking Caribbean region	Studies including samples of Caribbean immigrants and non-English speaking Caribbean states
Study design and publication type	Empirical studies with any research design (e.g., quantitative, qualitative, mixed methods)	Reviews or other study designs, or studies with non-human participants were excluded. Articles not published in English or publications containing insufficient extractable information were also excluded. Theses, book chapters, commentary articles, editorials, conference abstracts or protocols were not included in their current state but instead were trolled to identify associated peer-reviewed published articles for inclusion

### Appraisal of sources of information

As recommended by established scoping review guidelines [34, 37], a formal quality appraisal or risk of bias evaluation of the cumulative body of evidence was not conducted. In the absence of this we acknowledged peer reviewed articles as a measure of overall good quality, as done in previous scoping reviews [44].

### Data charting

Researchers with significant experience in CYP mental health practice, research, policy and lived experience experts provided input to reach a consensus regarding final data to be included. Data from each study were systematically extracted onto a matrix table. Data included reference, year, country, study aims, study design, sample size, setting, clinical characteristics, key findings, type of support, outcome, and descriptive characteristics of the sample and any intervention. When available, data on funding sources were also extracted. At least two reviewers independently extracted data. Any discrepancies were discussed, and, if necessary, a third member of the team was consulted to reach a final decision.

### Consultation exercise

In order to reach CYP mental health experts from different Caribbean islands, virtual consultations were undertaken. The consultation was designed primarily to inform and validate findings from the review. However, it was necessary to obtain input at earlier stages to sensitise the primary reviewer to issues that may or may not appear in the literature; and signpost the reviewer towards relevant studies. Representatives from a range of organisations, including policy makers, researchers and practitioners were consulted. Professionals either expressed interest in the study after coming in contact with the study protocol or through the reviewers’ own network. We also aimed to capture the views of parents and carers and young people with lived experience of mental health problems. Lived experience experts were known to at least one member of the review team and volunteered to participate after informal discussions about the purpose of the study. Consultations were conducted individually or in groups, and notes were taken solely to inform the study process. Although formal ethical approvals were not required, the consultation exercises were conducted in line with established ethical codes of conduct [45].

### Summarizing and mapping the evidence

The extracted data was collated and summarized to produce a descriptive narrative and numerical account of the included literature [46]. Regular meetings and email communication were used to discuss and cross-check the findings to avoid any risk of bias. To address the research aims, the findings were organised according to the characteristics of the reviewed studies, the psychosocial characteristics of the samples (e.g., externalising problems) and the overview of interventions and outcome measures. Subsequently, the findings from the review of the literature and the consultation activities were summarised to highlight the common research limitations and knowledge gaps.

## Results

**Fig. 1:**
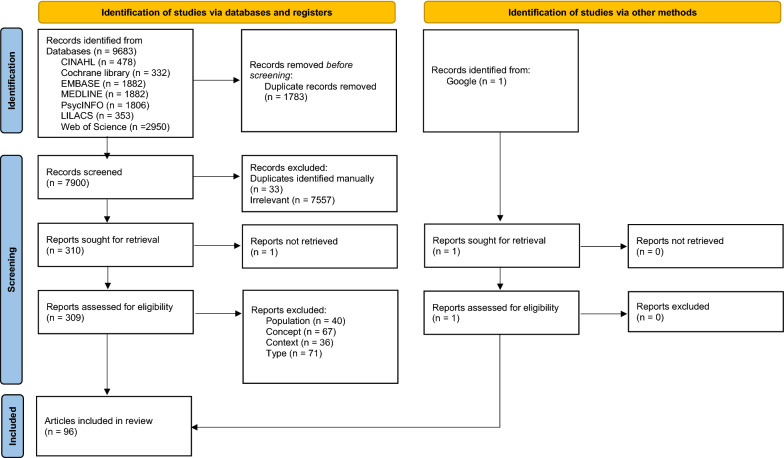
PRISMA flow diagram

The comprehensive search yielded 7901 records. After duplicates were removed, the remaining records were screened based on the title and the abstract and 7557 records were excluded. Attempts were then made to retrieve the remaining 311 full text articles. Of these, 215 were excluded based on population (k = 40), concept (k = 67), context (k = 36), study or publication type (k = 71) and availability (k = 1). After the searching and screening process, 96 records were deemed relevant to be included in the review. The PRISMA flow diagram presented in Fig. 1 provides step-by-step details of the study selection process.

### Characteristics of the findings

Articles were published from 1976 to 2020 with just about half of the reviewed articles (50, 52.08%) being published in the last decade. Most of the articles described studies conducted in Jamaica (k = 51, 53.13%), followed by Trinidad and Tobago (k = 18, 18.75%), Barbados (k = 8, 8.33%), Guyana (k = 4, 4.17%), Bermuda (k = 2, 2.08%), St. Vincent and the Grenadines (k = 2, 2.08%), St Kitts and Nevis (k = 2, 1.04%), St Lucia (k = 1, 1.04%) and The Bahamas (k = 1, 1.04%). Eight (8.33%) articles reported studies that collected data from multiple Caribbean countries. Most of the articles adopted quantitative research methods (k = 90, 93.75%) of which only three randomised controlled trials were reported. Three (3.13%) articles were qualitative studies and three (3.13%) were mixed methods designs. Sample sizes for the studies varied, ranging from 1 to 15,695 participants. Only twelve studies (12.5%) had sample sizes with less than fifty participants. Participants were predominantly recruited in education settings (k = 75, 78.13%). Fewer studies recruited participants in healthcare (k = 13, 13.54%) and community (k = 8, 8.33%) settings. Funding for the research was generally sparse, with only 36 (37.5%) out of the total 96 articles acknowledging sources of funding either from government, charities, or academic institutions locally, regionally or internationally.

The articles mainly focused on emotional distress, including depression and suicide issues (40, 41.67%), followed by behaviour and conduct problems (k = 19, 19.79%), eating and body image issues (k = 6, 6.25%), and personality (k = 3, 3.13%). Twenty-two articles (22.92%) focused on multiple problems, including both internalising and externalising problems, and five articles (5.21%) referred to any mental health problems that CYPs may experience. Age ranged from 2 to 24 years with a smaller number of studies (k = 12, 12.5%) focusing on younger children with mean, mode or upper age limit under age twelve. Fifteen (15.63%) articles focused on older young people with mean, mode or lower age limit between eighteen and twenty-four. Sixty-nine articles (71.88%) included adolescent samples with mean, mode or lower age limits between 12 and 17. For three of the articles the ages were not explicitly stated, but the authors described the samples as preschool-aged children, adolescents or university undergraduate samples. A little over half of the reviewed articles (k = 55, 57.29%) described samples that were fairly-equally distributed in terms of sex. Twenty-six articles (27.08%) had samples with over 60% females compared to nine (9.38%) articles which included samples with over 60% males. Six articles (6.25%) did not provide sufficient details to ascertain the sex of the participants. Table 2 provides a numerical summary of the key information. Further details of the characteristics of the reviewed articles (K = 96), sorted in descending order by the year of publication is presented in Additional file 2.

**Table 2:** Summary of the reviewed articles

Characteristics of the reviewed articles	Number of articles	% of articles	Cumulative %
Year of publication			
1976–1989	6	6.25	6.25
1990–1999	9	9.38	15.63
2000–2009	31	32.29	47.92
2010–2020	50	52.08	100
Country			
Jamaica	51	53.13	53.13
Trinidad and tobago	18	18.75	71.88
Barbados	8	8.33	80.21
Guyana	4	4.17	84.38
St Vincent and the Grenadines	2	2.08	86.46
Bermuda	2	2.08	88.54
The Bahamas	1	1.04	89.58
St Kitts and Nevis	1	1.04	90.63
St Lucia	1	1.04	91.67
Multiple	8	8.33	100
Study design			
Qualitative	3	3.13	3.13
Mixed methods	3	3.13	6.25
Quantitative	90	93.75	100
Area of focus			
Depressive symptoms (including stress, loss, PTSD, emotional and psychological distress)	25	26.04	26.04
Behavioural and conduct problems	19	19.79	45.83
Suicidality	15	15.63	61.46
Disordered eating and image issues	6	6.25	67.71
Personality	3	3.13	70.83
Anxiety	1	1.04	71.88
Any	5	5.21	77.08
Multiple	22	22.92	100
Recruitment settings			
Education	75	78.13	78.13
Healthcare	13	13.54	91.67
Community	8	8.33	100
Sex of participants			
Majority males (> 60%)	9	9.38	9.38
Majority females (> 60%)	26	27.08	36.46
Fairly evenly distributed (50–60% male and female)	55	57.29	93.75
Not clearly stated	6	6.25	100
Age of participants (years)			
Under 12	12	12.50	12.50
12–17	69	71.88	84.38
18–24	15	15.63	100.00
Sample size			
Small (< 50 participants)	12	12.50	12.50
Medium (50–300 participants)	32	33.33	45.83
Large (over 300 participants)	52	54.17	100
Source of funding			
Government	3	3.13	3.13
Academic institutions	10	10.42	13.54
Other	11	11.46	25.00
Multiple	12	12.50	37.50
None reported	60	62.50	100

### Internalising and externalising behaviours

Together several studies investigated internalising problems such as depression, stress, emotional distress, eating issues, anxiety, sadness and suicidality [26, 47–92], and externalising problems such as behaviour and conduct problems [93–111]. Some studies also focused on personality [112–114] or multiple problems [23, 115–140]. Most of these studies explored prevalence or risk and protective factors associated with these problems. Some articles examined the identification of psychosocial difficulties, the support CYP received, and fewer studies also explored views, attitudes, and experiences of various mental health problems. Some articles also examined how teachers and parents rated the problem.

The findings from the reviewed studies indicated prevalence and incidence rates ranging from 4.5 to 62% for internalising behaviours [61, 69] and 21–90.9% for externalising behaviours in CYP [115, 131]. Problems with relationships, home or family [48, 84, 117, 123, 139] or the community [26, 47] and exposure to violence [96] were identified as key risk factors for mental health and wellbeing problems in CYP. Protective factors included religious affiliations [58, 75, 79], living with fathers [88] and academic achievement [68, 85].

Albeit individual preferences, teachers, family members, faith leaders and friends were identified as sources of psychosocial support [126]. Although family members were ranked as a primary source of support, parenting styles, attachment and the child receiving physical punishment were also reported as influencing factors to the problematic behaviours in other studies [60, 81, 123]. The views and experiences of parents were not fully captured in any of the reviewed studies. However, professionals generally described the actual or perceived process of working with CYP with mental health problems as challenging [48, 119], while CYP focused on the lack of available support and stigma [116, 122]. Notably the interrater ratings of behaviour problems varied across studies. For example, there was low to moderate correlation between teachers and parents, and observers rated more behaviour problems than teachers [107, 109, 133, 137].

### Interventions

Among the reviewed articles four interventions were identified. One study evaluated the *IRIE Classroom Toolbox*, a school-based violence prevention programme [93]. Primary school teachers were trained on how to use positive and proactive strategies to promote children’s positive behaviours and prevent negative behaviours (e.g., through use of praise and teaching classroom rules). Children between 6 and 12 years were targeted to receive the intervention. Although teachers in the intervention schools used significantly less violence against children and provided a more emotionally supportive environment, benefits to class-wide child aggression, class-wide prosocial behaviour and child mental health were not significant. Another intervention identified was the *Function-based intervention*, a behavioural management tool, which was tested in a case study [105]. The intervention involved social skills and appropriate language training. The intervention was found to reduce the use of profanity and increase positive replacement behaviour in a 16-year-old girl.

The third intervention was based on the *Incredible Years Programme* [100, 104]. The intervention aimed to develop positive relationships with children and prevent and reduce inappropriate behaviours. Teachers initially received about 7- or 8-days training in the form of workshops on how to implement the strategies. In one study, when compared to a control group, the intervention significantly reduced children’s conduct problems, hyperactivity and peer problems in a group of preschool children but no significant benefits for prosocial and emotional problems [104]. In a similar study, the children attending preschools that participated in the Incredible Years programme showed significantly reduced conduct problems and increased friendship skills [100].

The modified *Dream-A-World* multimodal intervention aimed at promoting social adaptation and resilience using cultural therapies was also identified [99]. Students engaged with the intervention over two years and 6 months which incorporated workshops, field trips, after school skills training as well as the cultural therapy component. The group of 8–9-year-olds who participated in the intervention experienced significant changes in their behaviour, with overall improvements in aggressive, oppositional defiant behaviour and attention-deficit/hyperactivity problems as compared to the control group. These changes were also significant within-group from pre- to post-intervention. Notably, improvements were significant for boys whereas girls only improved marginally.

### Outcome measures

Fourteen (14.58%) studies used purposive author-developed or adapted outcome measures, while nine (9.38%) of the reviewed articles used the Beck Depression Inventory-II. A fair number of studies also used data collected through clinical interviews using the Diagnostic Statistical Manual (9, 9.38%). Other commonly used measures were the Global School Health Survey (7, 7.29%), Rosenberg Self-Esteem scale (5, 5.21%), Jamaican Youth Checklist (5, 5.21%), Eating Attitude Test (4, 4.17%), Massachusetts Youth Screening Instrument- Version 2 (3, 3.13%), Patient Health Questionnaire (3, 3.13%) and the Trinidad and Tobago Youth Survey (3, 3.13%). Notably some authors reported using a modified or adapted version of the Social Adjustment Scale and General Health Questionnaire because the items were not always culturally appropriate [83, 135]. However, only the Beck Depression Inventory-II [74], Brief Screen for Depression [76], Minnesota Multiphasic Personality Inventory [114], Trinidad and Tobago Youth Survey [98, 101], and the Jamaican Youth Checklist (teacher-reported) [108] were validated and revealed evidence for reliability and concurrent and discriminant validity in a Caribbean sample.

### Outcomes from the consultations

We obtained input from nine stakeholders. Four of whom were considered lived experience experts and included two parents and two young people. One of the parents identified as the father of a 14-year-old male with symptoms of depression and anxiety. The second parent identified as a mother of a 12-year-old girl with symptoms of depression and of a 17-year-old boy with diagnosed ADHD and conduct problems. Of the two young adults, one was a 21-year-old female attending university with a history of low mood and depression and one was a 24-year-old unemployed male with a history of conduct problems. For confidentiality purposes we are unable to report the geographical locations of the lived experience experts. The remaining five consultants worked in government (BM), academia (JE, AR-R) or local communities (PM, SG) in Barbados and Trinidad and Tobago. At the time of this study, two of the consultants were employed in the USA and the UK. Together, these professional consultants hold qualifications in social work, psychology, counselling and psychotherapy. Their roles varied involving policy development, therapeutic practice, research, or training in mental health. Outcomes and impact from the expert consultations are reflected in the discussion section of this article. The consultation exercises resulted in discussions that helped: (1) prioritise indicators to establish a research agenda, (2) establish learning materials to be included in future curriculums, (3) inform system, policy, or practice recommendations, and (4) develop a plan to inform knowledge translation and uptake strategies for the outcomes of this review.

### Indicators of evidence gaps and limitations

Although, this study acknowledges and commends the efforts of the authors of the reviewed studies, several central research gaps are prominent. Even with the use of solid designs, research participants were often at minimal risk of a disorder or of low severity, as recruitment was mainly done in schools and education settings. Methodologically, based on the number of studies conducted in recent years, and the few RCTs identified, more rigorous and high-level analytic qualitative and quantitative inquiry is also required to advance the field. The limited number of interventions required extensive training and outcomes did not focus on the longer term. This indicated there was a dearth of data on ongoing and follow-up data collection, making longitudinal studies essential. Further, several understudied areas were identified. In the area of CYP mental health, studies that focus on specific subpopulations (e.g., children below age 5), specific diagnoses (e.g., anxiety and psychosis), underrepresented countries (e.g., Anguilla), commonly underserved groups (e.g., sexual minority samples and children with physical disabilities) and complex mental health cases were lacking. It was also noted that the grey literature search was a limited resource for identifying appropriate material that led to peer-reviewed research evidence (n = 1).

## Discussion

As the first step to understand the current state of CYP mental health research in the English-speaking Caribbean region, this scoping review and evidence mapping exercise identified and summarised a range of studies (K = 96) exploring internalising and externalising problematic behaviours. Studies were published between 1976 and 2020. Most of the evidence came from quantitative studies conducted in Jamaica, Barbados and Trinidad and Tobago. Four interventions were identified for further development and evaluation. Albeit validation, most commonly used outcome measures were author-developed/adapted questionnaires. However, some evidence of psychometric validity was available for the Beck Depression Inventory-II, Brief Screen for Depression, Minnesota Multiphasic Personality Inventory, Trinidad and Tobago Youth Survey, and the Jamaican Youth Checklist. Several areas, such as, including data from younger children (< 12), a need for more funding opportunities, diversity in research designs, and information on CYP with severe clinical needs or under-represented/underserved groups were identified for further investigations.

### This review in the context of the broader literature

The current findings build on previous reviews by Brown et al. [27] and Pilgrim and Blum [28] which were based on fewer studies, specific age groups, and focused on specific disorders like depression and suicide behaviours. Yet the proportion of research (i.e., 50) we identified that had been conducted within the last decade is still not ideal when considering the international evidence suggesting an increase in internalising and externalising problems in CYP in the twenty-first century [141]. Our findings also add to the mixed views on sex differences on willingness to participate in research (e.g., [142]) by suggesting slightly more research activity among females when compared to males. However, this is still an understudied area in the CYP literature and may also indicate the influence of culture on the participants’ perception of research which could influence their decision to participate. Nonetheless, the extent and range of the literature appears to be comparable to similar regions [143] and adds to the broader evidence base for low-and-middle-income countries and developing nations [16, 144]. Similar to previous reviews [5–7], the current findings also highlight the relationships between CYP’s mental health and individual, familial and societal factors. Interestingly, when compared to the international evidence, the reviewed studies placed greater emphasis on spirituality and religion as a key protective factor for CYP mental health.

The importance of further research is also clear from the large number of quantitative descriptive studies and fewer randomised controlled trials. Based on the research pyramid and levels of evidence [145], more research is needed to facilitate evidence-based practice in the field of CYP’s mental health. The predominance of studies being conducted in schools and education institutes also aligns with researchers’ recommendations that schools can be a valuable resource for recruitment of participants for research involving children, adolescents, and parents [146]. This review further strengthens that recommendation with the considerably large enough samples sizes recorded in most of the reviewed studies. Despite the perceived willingness for the public to participate in research, the funding to carry out such research appeared to be limited. Based on the current findings, this may indicate a need to revisit or set a new agenda for research funding in the Caribbean region specifically to focus on CYP’s mental health [147].

The overwhelming number of studies focusing on depressive symptoms and behaviour problems among adolescents could be justified based on earlier studies that suggested the high prevalence of these problems among adolescents in the Caribbean [28]. Nonetheless this emphasis on depressive symptoms and behaviour problems could indicate a significant gap in knowledge for other mental health problems like anxiety which is highlighted in the international literature as a common problem for CYP [6]. Similarly, more research is needed on complex cases, such as comorbidities and problems experienced by marginalised groups, such as lesbian, gay, bisexual, transsexual and queer plus (LGBTQ +) groups, migrant populations and young people living with disabilities or in poverty. Knowledge about how young people of different ages, and their caregivers, access different types of support for their mental health is also limited in the reviewed studies. This may indicate that more research is needed to pool the available evidence in this area to provide a broader outlook. Although the research explored both internalising and externalising problems, the obvious lack of promising interventions calls for the development and evaluation of new interventions. That said, there are numerous psychotherapeutic [17, 148] and pharmacological [149] interventions, and a variety of modes of delivery [20] that could be considered for adaptation and delivery in the Caribbean.

The large number of studies conducted in Jamaica and Trinidad and Tobago can be attributed to the fact that these two islands are among the most populated of the English-speaking Caribbean with approximately 2.7 and 1.3 million people respectively [150]. This pattern was also observed in other reviews [27, 28]. However, this may not provide insight into whether the problems are more prevalent in these countries. Therefore, further research making the necessary subgroup comparisons is needed. In this same vein, it was important to note the five culturally validated outcome measures. The limited number of measures may suggest the necessity to identify, adapt and further validate outcome measures for use in this population. These activities could build on existing knowledge [151, 152] which suggest the need for outcome measures that are designed specifically for younger populations, feasible for use in routine care, and that are culturally adaptable.

### Future directions, implications and recommendations

In order to respond to the needs of stakeholders, the consultation exercises identified priorities and questions to guide future research, with agreement that the current evidence base could inform the line of questioning. First, it was recommended that further explorations of the limitations of the reviewed studies and a more in-depth analysis of the prevalence and risk factors could benefit the advancement of the field (PROSPERO CRD42021283161). More primary research is also needed in Caribbean countries less represented in this scoping review (e.g., Anguilla and St Lucia). Further, given the lack of research in some areas (e.g., anxiety and psychosis), mixed-methods approaches will be valuable to explore CYP’s, parents’, teachers’ and primary care practitioners’ perspectives and quantify the extent to which barriers influence management, and ability to identify these problems. These findings can then be used to target strategies to improve access to good-quality mental health care. Future research should also aim to develop and validate measurements that are more robust to minimise instances where researchers are forced to used single-item questionnaires or author developed or adapted measures that may lack psychometric validity.

In light of the limitations of this review, the current evidence base does not yet clearly indicate what intervention (s) or intervention components are most effective for meeting the needs of CYP in the Caribbean. Thus, professionals who work with families with children or young people are encouraged to be aware of the limitations of the evidence when selecting services and interventions for CYP experiencing mental health difficulties. This is an important consideration as experts consistently highlight the importance of cultural adaptions in patient care and treatment [17, 19]. Along with informing mental health practitioners, literature from the CYP’s mental health field points to the importance of disseminating knowledge to teachers, general practitioners and social workers. The most obvious improvement is the need for more interventions to provide services for prevention and treatment of mental health concerns. Similarly, developing or tailoring appropriate and evidence-based screening tools for common mental health problems, as already exists in the western world, would be another next positive step.

This review may also begin to inform conversations around disparities and inconsistencies in CYP mental health. For example, behavioural problems and depressive symptoms remain prevalent, yet with little or no focus on suitable interventions. It is also important for policymakers and practitioners to consider the emotional well-being of parents of children with mental health disorders and teachers who work with these groups, as they may also require support for their own mental health whilst caring for the child. Therefore, this scoping study can aid in an attempt at highlighting a critical need for resources. The lack of resources may suggest increased funding or the better use of funding to evaluate and explore new ways to optimize the availability of resources for stakeholders. Consequently, the knowledge gained from this review may be of interest to policy makers locally, regionally and internationally.

This review also clearly identified a lack of high-quality rigorous research activities (e.g., randomised controlled trials). This may be reflective of a lack of training in this area resulting in a serious skill gap. The further development and promotion of appropriate courses would be a positive step to rectify this situation. Lastly, it is also recommended that the general public receive the necessary public health awareness messages to understand mental health challenges and access the available resources for CYP.

### Strengths and limitations of this study

This review adhered to established guidelines for scoping reviews (see Additional file 3) and adopted a comprehensive search strategy including grey literature searches. At least two independent reviewers searched, screened, identified and extracted relevant information and a third reviewer was brought in whenever disagreements occurred, strengthening the objectivity of the process. However, as with most reviews, there is a possibility that some relevant information could have been unavoidably missed during the searching and screening process. Further, only studies published in English were included in this review. Therefore, literature published in other languages may have been excluded. This also aligns with the decision to focus only on the evidence from English-speaking Caribbean countries. Notwithstanding the importance of CYP’s mental health in the broader region, we believe that although findings may be transferable, a narrow focus is needed before we can expand to the wider Latin America and Caribbean region. Another possible limitation could be that the searches were mainly conducted online, which would not capture offline material. It is also noted that some islands were under-represented both in this review and the consultation exercises, therefore attempts to generalise the findings should be taken with caution. Lastly, although this review was an enormous undertaking, the results are only up to date as of January 2021.

## Conclusion

This scoping review reports a fairly large number of studies providing insight on useful, but sometimes limited range, of scientific evidence. Accumulating evidence suggests a wealth of knowledge on the prevalence of socioemotional and behaviour problems. However, there seems to be a limited number of studies focusing on problems like psychosis and anxiety and a lack of investigations around complex cases and other vulnerable groups. There is also a need for further studies aimed at developing and evaluating more innovative interventions and outcome measures. Given the prevalence and significance of the issue, we hope academics, practitioners, and decision-makers may use this review as a preliminary guide to inform future activities to support CYP’s mental health in the Caribbean region.

## Supplementary Information

**Additional file 1. **Sample search strategy.

**Additional file 2. **Characteristics of the reviewed studies (K=96).

**Additional file 3. **Preferred Reporting Items for Systematic reviews and Meta-Analyses extension for Scoping Reviews (PRISMA-ScR) Checklist.

## Data Availability

Not applicable.

## Abbreviation

CYP: Children and young people
