# Supplementary material for: Children and young people’s mental health in the English-speaking Caribbean: a scoping review and evidence map
Source: Child Adolesc Psychiatry Ment Health. 2021 Dec 30;15:82. doi: 10.1186/s13034-021-00435-w (PMC8719385; doi:10.1186/s13034-021-00435-w)
Supplement: Supplementary file 1 — Additional file 1. Sample search strategy. [file 13034_2021_435_MOESM1_ESM.pdf]

Supplementary Information 1. Sample search strategy

Concept	Search string
Children and young people	child or youth or "young people" or adolescent or teen or preteen or kid or tween or pediatrics or pupil or student or "young adult" [Title/Abstract]
Mental health and wellbeing	"mental health" or "well being" or "mental disorder" or "mental problem" or anxiety or "emotional states" or "anxiety disorder" or "general anxiety disorder" or "panic disorder" or "panic attack" or phobias or "posttraumatic stress" or shame or stress or depression or sadness "attention deficit disorder" or "oppositional defiant disorder" or "behavior disorder" or "conduct disorder" or "disruptive behaviour disorder" or "impulse control disorder" or "oppositional defiant disorder" or "self destructive behaviour" or "aggressive behavior" or "antisocial behavior" or "behaviour problems" or " externalizing symptoms" or "personality disorders" or personality or emotions or "emotional health" or " negative emotions" or "intellectual development disorder" or "autism spectrum disorders" or "behaviour problems" or "antisocial personality disorder" or psychopathy or "antisocial behaviour" or "anxiety disorder" or schizophrenia or psychosis or bipolar or suicide or "self-harm" or "adolescent psychology" or psychiatry or development or "child psychiatry" [Title/Abstract]
Caribbean	Caribbean or "greater antilles" or "lesser antilles" or "leeward island" or "windward islands" or "west indies" or Trinidad or Tobago or Antigua or Barbuda or Barbados or Martinique or "Dominican Republic" or Haiti or Jamaica or cuba or bahamas or dominica or " saint lucia" or grenada or guadeloupe or curacao aruba or "netherlands antilles" "US Virgin Islands" or "british Virgin Islands" or "Saint kitts" or Nevis or "sint maarten" or "saint vincent" or grenadines or hispaniola or saba or "virgin islands" or tortula or "Virgin Gorda" or Anegada or " Saint Crix" or "saint thomas" or "sombrero or "saint martin" or "cayman island" or montserrat or "turks and caicos" or anguilla or beliz or bonaires or bermuda or guyana or suriname [Title/Abstract]
