# Supplementary material for: Children and young people’s mental health in the English-speaking Caribbean: a scoping review and evidence map
Source: Child Adolesc Psychiatry Ment Health. 2021 Dec 30;15:82. doi: 10.1186/s13034-021-00435-w (PMC8719385; doi:10.1186/s13034-021-00435-w)
Supplement: Supplementary file 2 — Additional file 2. Characteristics of the reviewed studies (K=96). [file 13034_2021_435_MOESM2_ESM.pdf]

Supplementary Information 2. Characteristics of the reviewed studies (K=96)

Reference: First author, year of publication	Country	Study aim(s)	Presenting problem explored	Outcome measures	Study design	Sample size	MeanAge (SD) or age range in years	Predominant sex (%)	Primary recruitment setting	Funding source	Key finding(s)
Griffith, 2020	Barbados	To examine how teachers identify and respond to social-emotional, behavioural, and learning challenges.	Multiple: socioemotional and behavioural challenges	Author developed/adapted questionnaire	Quantitative	90	2 to 7	Unclear	Education	None reported	Teachers on average reported one-third of children in their classes had significant socioemotional and behavioural challenges. Few teachers reported using screening measures to assess for these difficulties, and teachers generally reported that their training in identifying young children with socioemotional, behavioural or learning difficulties was inadequate.
Harrison, 2020	Jamaica	To explore disordered eating and identify those adolescents most at risk.	Disordered Eating Behaviours and Attitudes	Eating Attitude Test (EAT); Rosenberg Self-Esteem (RSE) scale	Quantitative	521	14.8 (not specified)	56.05% females	Education	Caribbean Public Health Agency (CARPHA).	31% percent of participants reported engaging in at least one disordered eating behaviour, with bingeing as the most common. Female participants had significantly higher mean body mass index and mean EAT-26 score compared to males.
Maloney, 2020	Jamaica	To examine digital mental health (mHealth) infrastructure in Jamaica; receptiveness to mHealth resources; identify barriers to treatment and treatment preferences; and measure treatment optimism and mHealth interest.	Any	Author developed/adapted questionnaire	Quantitative	163	10 to 19	Unclear	Community	None reported	Adolescents reported very few practical or infrastructure barriers to accessing digital mental health resources. Majority were interested in using mental health apps to monitor their own mental health and expected that digital solutions could benefit adolescents with symptoms of mental distress. Stigma, shame, and embarrassment were major barriers to help-seeking and professional help was only preferred for more severe conditions such as psychosis and substance abuse.
Chung, 2020	Jamaica	To investigate the association between children's perception of their communities and their levels of depressive symptoms.	Depressive symptoms	The Adolescent Depression Rating Scale (ADRS)	Quantitative	321	10 to 12	49.8% females (7.8% did not respond)	Education	None reported	There were significant associations between neighbourhood factors and depressive symptoms while neighbourhood factors and social class were predictive of children's depressive symptoms.
Gardner, 2019	Jamaica	To explore the associations of perceived family and peer social support, self-esteem, and psychological well-being.	Multiple: self-esteem, psychological well-being, stress, anxiety, and depression	Depression, Anxiety, and Stress Scale–Short Form (DASS-21); Psychological Well-being Scale–Short Version (PWB-18); Multidimensional Scale of Perceived Social Support; Rosenberg's Self-Esteem (RSE) Scale	Quantitative	334	14.56 (1.94)	51% males	Education	None reported	Family support showed direct and indirect associations with depression, anxiety, stress, and well-being through self-esteem while peer support only showed a direct positive association with well-being.
Johnson, 2019	Guyana	To explore the police officers experience in dealing with the adolescents who made a non-fatal attempt to commit suicide.	Suicidality	Not applicable	Qualitative	13	Adolescents	55.41% females	Community	University of the West Indies, St Augustine Campus	Police officers described four main issues which suicidal adolescents typically present: relationship problems, family conflict, conflicts regarding sexual orientation and peer pressure.
Baker-Henningham, 2019	Jamaica	To investigate the effect of a school-based violence prevention programme.	Behaviour and conduct problems	Observations; Emotional SupportDomain in the Classroom Assessment Scoring System (CLASS-K-3)	Quantitative	220	6 to 7	54% males	Education	Grand Challenges Canada (Global Mental Health Stream)	Intervention teachers provided more emotionally supportive classroom environment. No significant benefits were found toclass-wide prosocial behaviour, teacher wellbeing, or child mental health.

Elledge, 2019	Jamaica	To investigate whether bullying victimization and parental involvement were associated with internalizing distress, suicidal thoughts and behaviour, and substance use in adolescents as well as whether parental involvement moderated the relationship.	Multiple: suicidality, internal distress (worry, loneliness)	Jamaica Global School-based Health Survey (GSHS)	Quantitative	1623	11 to 16	51.2% females	Education	None reported	Bullying victimization was uniquely and positively associated with feelings of loneliness, sleep difficulties due to worry, smoking frequency, and suicidality for both male and female adolescents as well as with alcohol use frequency for female adolescents.
Bateman, 2019	Jamaica	To examine the psychological sequelae experienced by high-level athletes as a result of sport-related injuries based on a PTSD–self-efficacy framework.	PTSD	Impact of Event Scale-Revised (IES-R); General Self-Efficacy Scale (GSES)	Quantitative	46	18 to 24	65.2% males	Education	None reported	The presence of injury was a significant predictor of general PTSD and, specifically, hyperarousal symptoms; however, general self-efficacy was not found to predict trauma-related symptoms.
Gardner, 2019	Jamaica	To examine the interacting effect of trait emotional intelligence on the association of self-esteem and depressive symptoms.	Depressive symptoms	Depression, Anxiety and Stress Scale Short (DASS) Form; Rosenberg Self-esteem (RSE) Scale; Schutte Emotional Intelligence Scale	Quantitative	334	14.74 (1.95)	51% males	Education	None reported	Lower self-esteem and trait-emotional intelligence was associated with more depressive symptoms.
Descartes, 2019	Trinidad and Tobago	To examine whether self-esteem predicted aggression.	Behaviour and conduct problems	Direct and Indirect Aggression Scale (DIAS); Culture Free Self-Esteem Inventories (CFSEI-3)	Quantitative	170	9 to 15	52% females	Education	None reported	Global self-esteem was a stronger predictor of aggression for children than domain specific self-esteem. Domain specific self-esteem was a stronger predictor of aggression among adolescents.
Youssef, 2018	Trinidad and Tobago	To assess medical students' knowledge about and attitudes toward mental illness.	Any	Medical Conditions Regard Scale (MCRS); 2) Previously validated measure to asses knowledge (unknown).	Quantitative	356	24.7 (0.3)	64.04% females	Education	None reported	Students revealed significant levels of stigmatization toward mental illness despite improvements in knowledge. Students recognized the need to prioritize treatment for persons with mental illness but did not want to be personally involved in the treatment process.
Oshi, 2018	Barbados	To examine the role alcohol plays in students' behaviour problems at school.	Behaviour and conduct problems	National Secondary Schools Survey	Quantitative	8109	14 (not specified)	59% females	Education	None reported	Neither past month nor year alcohol consumption by students was associated with frequent behaviour problems at school. Students under 17 years who had a relationship with a teacher that was not very bad were significantly less likely to engage in frequent behavioural problems.
Debowska, 2018	Barbados & Grenada	To uncover groups of youths with specific abuseprofiles and identify how the abuse is associated with behavioural outcomes.	Behaviour and conduct problems	Author developed/adapted questionnaire	Quantitative	1351	9 to 17	50.1% females	Education	European Union	Compared with members of low abuse groups, youths who reported having experienced high/moderate levels of various forms of violence, including those who were abused in multiple ways and across the two settings, were significantly more likely to engage in violent and hostile behaviours.
Giray, 2018	Jamaica	To explore the quantity (frequency) and quality (priority, atmosphere, structure) of family mealtimes and associations with nutritional and emotional health in Jamaica.	Depressive symptoms	Jamaican Youth Risk and Resiliency Behaviour Survey, Patient Health Questionnaire (Adapted)	Quantitative	366	13.8 (not specified)	64% females	Education	University of Illinois	High SES adolescents ate more unhealthily if their mothers had more difficulty finding time for family meals. Adolescents and mothers were more psychologically distressed if they themselves had more difficulty finding time for family meals, if they had fewer positive attitudes/behaviours around mealtime atmosphere, or if they were high SES individuals placing lower importance on mealtimes.

Heron, 2017	Jamaica	To determine prevalence of suicidal behaviours and differences related to gender and alcohol use among Jamaican adolescents.	Suicidality	Suicidal Behaviours Questionnaire-Revised (SBQ_R)	Quantitative	3471	10 to 19	58.8% females	Education	United Nations Children's Fund	There was no significant direct association between alcohol use and suicidal behaviour. Females who used alcohol had higher suicidal ideation and suicidal risk scores than females who did not use alcohol. No significant differences between male alcohol users and non-users and suicidal behaviour.
Kwangu, 2017	Bahamas	To estimate the prevalence of suicidal ideation and determine its correlates among adolescents.	Suicidality	Global School-based student Health Survey (GSHS)	Quantitative	1357	13 to 17	53.1% females	Education	None reported	Suicidal ideation prevalence among adolescents was 18.2% (13.8% among males and 22.3% among females). Male gender was protective against suicidal ideation. Other variables were found to be risk factors for suicidal ideation, and these were; age below 16 years, anxiety, loneliness, bullied, physical fights, smoking cigarettes and parental understanding.
Siziya, 2017	Guyana	To monitor trends in the prevalence of health behaviours and establish protective factors.	Suicidality	Global School-based Health Survey (GSHS)	Quantitative	2392	Mode 14 to 15	51.5% female	Education	None reported	Suicidal ideation was reported by 23.5% (16.4% of male and 29.7% of female) participant. Correlates for suicidal ideation were age, gender, anxiety, loneliness, close friends, truancy, bullied, attacked, currently smoked cigarettes, alcohol abuse, current alcohol use and parental understanding.
Siziya, 2017	Jamaica	To determine prevalence of suicidal ideation as well as factors associated with suicidal ideation.	Suicidality	Jamaica Global School-based Health Survey (GSHS)	Quantitative	1623	Grade 7 to 12	51.4% males	Education	None reported	Overall,24.8% (23.0% of male and 25.8% of female) students reported suicidal ideation. Factors associated with suicidal ideation were age, gender, food security, anxiety, loneliness, close friends, truancy, bullied, currently smoked cigarettes, alcohol consumption [currently drank alcohol and alcohol abuse and parental understanding.
Denton, 2017	Guyana	To describe and identify risk and protective factor correlates of youth suicidal behaviour among those at highest risk for suicide – orphans.	Suicidality	DSM-5 Level 1 Cross-Cutting Symptom Measure; Behavioural Assessment Schedule for Children, 2nd Edition (BASC-2)	Mixed Methods	25	12.7 (not specified)	52% females	Healthcare	None reported	Nine of the 25 (36%) orphans reported a previous suicide attempt. Youths who endorsed suicidal behaviour had clinically elevated interpersonal relations scale scores when compared to youth who did not.
Graham, 2016	Jamaica	To determine the levels of stress among students.	Stress	Author developed/adapted questionnaire	Quantitative	106	23.16 (9.0)	97.2% females	Education	None reported	Students enrolled at 2 nursing schools experienced moderately high levels of stress in the clinical environment. Financial concerns and interaction with ward staff were significantsources of stress.
Youssef, 2016	Trinidad and Tobago	To explore the prevalence of stress, burnout, and depressive symptoms and associated risk factors among medical students.	Multiple: depressive symptoms and stress	Maslach Burnout Inventory (MBI); Perceived Medical School Stress scale (PMSS); Patient Health Questionnaire (PHQ-9)	Quantitative	381	22.4 (3)	67% females	Education	None reported	Students demonstrated high levels of stress and a significant prevalence of burnout (52 %) and depressive symptoms (40%). Students who felt they lacked emotional support, had little opportunity for relaxation and exercise, and did not feel they had control of their daily schedule all demonstrated higher levels of burnout and depressive symptoms.
Maguire, 2016	Trinidad and Tobago	To examine family risk factors that impact adolescents' social decision making and behaviour.	Behaviour and conduct problems	Trinidad & Tobago Youth Survey (TTYS)	Quantitative	2552	15.4 (not specified)	60% females	Education	Government : Ministry of National Security of Trinidad and Tobago	Adult history of antisocial behaviour and parental attitudes were favourable toward antisocial behaviour and substance use emerge as the most robust risk factors.

Harrison, 2015	Jamaica	To determine the prevalence, risk and protective factors associated with disordered eating behaviours and attitudes.	Disordered Eating Behaviours and Attitudes	Rosenberg Self-Esteem (RSE) scale; EAT-26 scale; Parent and Peer Attachment Scales.	Quantitative	524	14.9 (not specified)	56% females	Education	Caribbean Public Health Agency (CARPHA)	23% of participants had scores $\geq$20 with bingeing being the most common. Higher scores were associated with females, having a history of sexual abuse and higher negative affect. Protective factors included living with their father.
Toussaint, 2015	Trinidad and Tobago	To examine religiousness and its connection to suicidal thoughts, plans, and attempts.	Suicidality	Trend Research Empowering National Development (TREND)	Quantitative	4448	18.14 (1.16)	57% females	Community	None reported	Compared to nonreligious, Catholics and Seventh-day Adventists were less likely to think about suicide, and Hindus and other affiliates were more likely to be treated for suicide. Higher self-rated religiosity was related to lower likelihood of thinking about suicide and lower likelihood of planning suicide. Attendance at religious services was related to lower likelihood of thinking about suicide and fewer suicide attempts. More frequent prayer was related to lower likelihood of thinking about suicide and lower likelihood of planning suicide.
Jackson Williams, 2014	Jamaica	To examine predictors of psychological help-seeking attitudes among adolescents.	Any	Attitudes Toward Seeking Professional Psychological Help scale (ATSPPHS); Opinions about Mental Illness scale; Brief Symptom Inventory (BSI)	Quantitative	339	15 to 19	57% females	Education	None reported	Increased age, decreased authoritarian beliefs, and increased benevolence predicted more positive attitudes toward seeking psychological help.
Lowe, 2014	Jamaica, Bahamas, St. Vincent and the Grenadines & St. Kitts & Nevis	To examine the association of perceived neighbourhood conditions with depressive symptoms.	Depressive symptoms	Beck Depression Inventory (BDI-II)	Quantitative	1955	15.3 (0.95)	52.1% females	Education	University of the West Indies	52.1% reported mild to severe depressive symptoms. Neighbourhood factors were more highly associated with depression in Jamaica than the other islands.
Wilson-Mitchel, 2014	Jamaica	To explore the experiences and the impact of pregnancy on adolescents psychologicalhealth.	Multiple: psychological distress or suicidality	Not applicable	Mixed Methods	30	15.6 (0.93)	100% females	Healthcare	International Developmen tResearch Centre (IDRC); Canada Latin America and the Caribbean Research Exchange GrantsProgr am (LACREG); Ryerson University	Themes emerged describing decision-making, resilience, social support, community support system, distress, and perceptions of services.
McFarlane, 2014	Jamaica	To assess the prevalence of depression and the associated risk factors in youths.	Depressive symptoms	Jamaica Youth Risk and Resiliency Behaviour Survey; The Ministry of Health screening tool (adaptation of the Diagnostic and Statistical Manual of Mental Disorders 4th Edition)	Quantitative	1312	15 to 19	54.57% females	Community	US Agency for International Developmen t	The results show that the prevalence of depression is high in this age group (males=9.7%, females=21.3%, total=15.5%).
Maguire, 2013	Trinidad and Tobago	To develop measures of family risk and protective factors for adolescent problem behaviours.	Behaviour and conduct problems	Trinidad and Tobago Youth Survey (TTYS)	Quantitative	2552	15.4 (not specified)	60% females	Education	Government : Ministry of National Security and	The measures have strong construct validity.

										Ministry of Education	
Smith, 2013	Jamaica	To examine the relationship between parenting style and adolescents' psychological and behavioural well-being.	Multiple: psychological and behavioural wellbeing	Massachusetts Youth Screening Instrument: Version 2 (MAYSI-2)	Quantitative	563	15.10 (not specified)	57.2% females	Education	University of Tennessee	Authoritarian parenting was associated with diminished psychological and behavioural adjustment, such that adolescents who reported their parents as more authoritarian also reported a greater risk of anger depression, suicide ideation, and conduct problems.
Guzder, 2013	Jamaica	To assess the effectiveness of a multimodal after school and summer intervention called the Dream-A-World (DAW) Project.	Behaviour and conduct problems	Achenbach System of Empirically Based Assessment (ASEBA) Teacher Report Form	Quantitative	30	9.1 (0.41)	57% males	Education	University of the West Indies, Mona Campus	The intervention group made significant improvements in school social and behaviour adjustment with more successful outcome amongst boys for behavioural gains. No significant improvements were made by the girls.
Jackson Williams, 2013	Jamaica	To compare attitudes towards seeking professional psychological services.	Any	Attitudes Toward Seeking Professional Psychological Help scale	Quantitative	279	17.18 (0.76)	56.63% females	Education	None reported	Jamaicans were generally found to be either similar or more positive in their attitudes toward seeking professional psychological help.
Smith, 2012	Jamaica	To examine the relationships among selected family interaction variables and psychosocial outcomes.	Multiple	Massachusetts Youth Screening Instrument Version 2 (MAYSI-2); Author-developed behaviour problem questionnaire.	Quantitative	171	15.14 (1.40)	54.2% males	Education	None reported	Family interaction variables (e.g., physical violence and verbal aggression) were positively associated with problematic adolescents' adjustment.
Baker-Henningham, 2012	Jamaica	To determine the effects of a universal pre-school-based intervention on child conduct problems and social skills at school and at home.	Behaviour and conduct problems	Not applicable	Quantitative	225	3 to 6	61% males	Education	Wellcome Trust Research Training Fellowship	A low-cost, school-based intervention in a middle-income country substantially reduced child conduct problems and increased child social skills at home and at school.
Jackson Williams, 2012	Jamaica	To examine adolescents' preferential sources of help for a variety of psychological problems and perceptions of the usefulness of seeking help.	Any	Author developed single item measure: Where Do You Go To For Help?	Quantitative	339	17.18 (0.76)	57% female	Education	None reported	Results of rank ordered data indicated that for many psychological problems, Jamaican adolescents were likely to first turn to friends and family for help. Faith healers, teachers and guidance counselors were consistently identified as the last sources of help among this population.
Lipps, 2012	Jamaica, Bahamas, St. Vincent and the Grenadines & St. Kitts and Nevis	To examine the association of parenting styles to levels of depressive symptoms among adolescents.	Depressive symptoms	Parenting Practices Scales (PPS); Beck Depression Inventory - II (BDI-II)	Quantitative	1955	15.3 (0.95)	52.1% females	Education	University of the West Indies - Mona	Nearly half (52.1%) of all adolescents reported mild to severe symptoms of depression with 29.1% reporting moderate to severe symptoms of depression. Authoritative and permissive parenting styles were both associated with lower levels of depressive symptoms in adolescents. The relationship of parenting styles to depression scores was not consistent across countries.
Abdirahman, 2012	Cayman Islands, St Lucia, St Vincent and the Grenadines, and Trinidad and Tobago	To examine the associations between peer victimization, mental health, and parental involvement.	Multiple: depression, anxiety, loneliness	Global School-based Student Health Survey (GSHS)	Quantitative	6780	13 to 15	52.98% females	Education	None reported	~25% reported having been bullied in the past month. Nearly 25% of students reported sadness and hopelessness, more than 10% reported loneliness and anxiety and more than 15% reported having seriously considered suicide in the past year.

Abel, 2012	Jamaica	To examine the prevalence of self-reported depressive symptoms, and the associated sociodemographic factors.	Depressive symptoms	Caribbean Adolescent Health Survey (PAHO); Global School Health Survey (WHO),	Quantitative	3003	12.45 (1.68)	52.65 % females	Education	None reported	4.5% reported depressive symptoms. Negative community attributes and protective factors within the home, gender, and learning problems were associated with depressive symptoms.
Holder-Nevins, 2012	Jamaica	To establish adolescent suicide rates for the years 2007-2010 and provide related epidemiological data.	Suicidality	Case notes	Quantitative	26	16 (3.01)	76.9% males	Community Police	None reported	The incidence for suicide in adolescents was 1.1 per 100 000. Most suicide cases were students and the majority of cases were from rural areas (65%). Hanging was the main method used to commit suicide (96.2%).
Williams-Johnson, 2012	Jamaica	To document the characteristics of self-poisoning suicide attempters.	Suicidality	Author developed/adapted questionnaire	Quantitative	127	Mode 16 to 20 and 56.7% under 25	75.6% females	Healthcare	None reported	The most common reason for suicide attempt was interpersonal conflict.
Abell, 2012	Jamaica	To determine the prevalence of suicidal ideation and examine the association between suicidal ideation and sociodemographic characteristics, protective and risk factors.	Suicidality	Global School Health Survey (WHO)	Quantitative	2997	10 to 15	52.69% females	Education	None reported	The prevalence rate of suicidal ideation was 9.7%. Correlates of suicidal ideation were being female, being depressed, living in a rural area, liking oneself, indulging in aggressive behaviour, considered harming others, protective factors in the home, involvement in risky behaviour and being teased/bullied.
Hull, 2011	Jamaica	To examine the tenability of a higher order factor structure of personality with Jamaican adolescents and young adults.	Personality	NEO-Five Factor Inventory	Quantitative	1018	17 to 24	59% females	Community	Inter-American Developmen t Bank	A model with five first order factors and one higher order factor fit Jamaican data.
Maguire, 2011	Trinidad and Tobago	To examine the validity of the community risk and protective factor measures developed by researcher in the Communities that Care program.	Behaviour and conduct problems	Trinidad and Tobago Youth Survey (TTYS)	Quantitative	2552	15.4 (not specified)	60% females	Education	Government : Ministry of National Security Trinidad and Tobago	Existing measures of risk and protective factors have weak construct validity when applied to youth in Trinidad and Tobago.
Smith, 2011	Jamaica	To examine the relationship between physical punishment and socioemotional wellbeing.	Multiple	Massachusetts Youth Screening Instrument-2 (MAYSI-2); Rosenberg Self-esteem Scale	Quantitative	563	15.08 (not specified)	57.2% females	Education	University of Tennessee	Physical punishment was significantly associated with adverse psychological and behavioural consequences.
Lipps, 2010	Jamaica	To investigate if students who attended non-traditional high schools would have higher levels of depressive symptoms than those in traditional high schools.	Depressive symptoms	Beck Depression Inventory–II (BDI-II)	Quantitative	278	15.0 (0.6)	52% females	Education	New Initiatives Grant	Students attending non-traditional high schools reported significantly higher depression scores than students in traditional schools.
Galler, 2010	Barbados	To examine the prevalence of depressive symptoms in youths with histories of infantile malnutrition.	Depressive symptoms	Minnesota General Adjustment and Morale Scale	Quantitative	177	11 to 17	Unclear	Education	National Institute of Health	Childhood malnutrition was associated with impaired cognitive and behavioural outcomes. Depressive symptoms were elevated in 11–17-year-olds with histories of malnutrition in the first year of life relative to healthy classmates.
Alleyne, 2010	Barbados	To examine stress and life satisfaction among university students.	Multiple: stress and life satisfaction	Satisfaction with Life Scale (SLS); Perceived Stress Scale (PSS)	Quantitative	172	24.9 (not specified)	70% females	Education	None reported	Higher levels of perceived stress were associated with lower levels of satisfaction with life.

Kukoyi, 2010	Jamaica	To conduct a preliminary investigations of risk factors associates with suicide ideation and attempt among youths.	Suicidality	9-country Caribbean survey (Adapted)	Quantitative	342	10 to 19	52.2% females	Education	National Centre on Minority Health and Health Disparities; National Institutes of Health	History of self-violence, violent thoughts toward others, mental health diagnoses other than depression, and a history of sexual abuse were positively associated with suicide attempt. Sexual abuse, mental health diagnoses other than depression, self-violence, and ease of access to lethal substances/weapons were positively associated with suicide ideation.
Lipps, 2010	Jamaica, St. Vincent and the Grenadines & St. Kitts and Nevis	To examine the levels of depressive symptoms among students.	Depressive symptoms	Beck Depression Inventory II (BDI-II)	Quantitative	1738	12 to 19	52% females	Education	None reported	Roughly half (53%) of the students reported some symptoms of depression with 19.2% reporting moderate and 10.7% reporting severe symptoms of depression. Students in Jamaica reported significantly higher depression scores than those in either St. Kitts and Nevis or St. Vincent and the Grenadines. Students assigned to a higher academic track reported significantly lower depression scores than students who were assigned to the lower academic track.
Jimerson, 2009	St. Vincent and the Grenadines	To examine peer status, academic scores and teacher-ratings of delinquency, learning problems, conduct and sensitivity.	Behaviour and conduct problems	Revised Behaviour ProblemChecklist (RBPC)	Quantitative	168	9.54 (2.04)	56% males	Community	Spencer Foundation; Mellon Foundation;Johann Jacobs Foundation; Sigma Xi Grantin-Aid; NIDA centre grant	Children who were considered sensitive andwithdrawn by their teachers, children who were considered delinquent and antisocial and older children had lower academic scores. Rejected and controversial children were also rated as having more learning problems than the other children.
Maynard, 2009	Barbados	To examine the coping strategies of problem adolescents.	Behaviour and conduct problems	Not applicable	Qualitative	20	14 to 16	50% males	Education	None reported	Coping strategies were identified as problem-focused, emotion-focused, support seeking and avoidance coping.
Lowe, 2009	St. Kitts & Nevis	To examine levels of depression.	Depressive symptoms	Beck Depression Inventory- II (BDI-II)	Quantitative	744	15.5 (0.8)	50.4% females	Education	None reported	62.1% (6 in 10) students reported some symptoms of depression. 14.8% reported moderate to severe and 9.7% reported severe symptoms.
Lowe, 2009	Jamaica	To examine the factors associated with depression.	Depressive symptoms	Brief Screen for Depression (BSD: 16)	Quantitative	690	23.4 (7.4)	77% females	Education	None reported	4 in 10 students (39.1%) scored in the clinically depressed range. Females reported significantly higher levels of depressive symptoms.
Baker-Henningham, 2009	Jamaica	To evaluate the effect of a preventative intervention in Jamaican schools on child behaviour and parent-teacher contacts.	Behaviour and conduct problems	Strengths and Difficulties Questionnaire (SDQ)	Quantitative	135	Pre-schoolers	65.19% males	Education	Environmental Foundation of Jamaica; UNICEF	The intervention had significant benefits for conduct problems, hyperactivity and peer problems.
Dookeran, 2009	Barbados, Jamaica, Trinidad and Tobago	To determine whether dissatisfaction with one's body was associated with unhealthy behaviours.	Multiple: disordered eating, depression	Eating Attitudes Test (EAT-26); Body Shape Questionnaire (BSQ-16); Body Silhouette Chart; Rosenberg Self-Esteem Scale (RSE); Centre for Epidemiologic Studies depression scale (CES-D)	Quantitative	383	21 (2.8)	51.96 males	Education	None reported	4.2% of participants reported to have been diagnosed with an eating problem. Females had significantly higher problem scores than males.

Maharaj, 2008	Trinidad and Tobago	To determine the prevalence of depression and psychosocial factors associated with depression.	Depressive symptoms	Beck Depression Inventory (BDI-II)	Quantitative	1290	13 to 19	58.2% females	Education	None reported	The prevalence of depression was 25.3%. Females, adolescents not living with both parents and adolescents afraid of their parents or of being injured by parents were more likely to be depressed.
Turton, 2007	Bermuda	To examine the effectiveness of function-based intervention with an adolescent girl with behavioural disorders.	Behaviour and conduct problems	Not applicable	Qualitative	1	16-year-old	100% females	Education	Not reported	A function-based intervention was effective in reducing problem behaviours and increasing replacement behaviours.
Rudatsikira, 2007	Guyana	To estimate the prevalence and associated factors of suicidal ideation.	Suicidality	Guyana Global School-Based HealthSurvey (GSHS)	Quantitative	1197	14 (not specified)	51% females	Education	None reported	18.4% (14.9% males and 21.6% females) reported having seriously considered committing suicide in the last 12 months. Males were less likely to seriouslyconsider committing suicide than females. Subjects who reported having been bullied were more than twice as likely to contemplate committing suicide. History of depression was positively associated with suicidal ideation while having close friends and understanding parents were negatively associated with suicide ideation.
Ekundayo, 2007	Jamaica	To determine the prevalence of depressive symptoms and its' associated factors.	Depressive symptoms	Beck's Depression inventory II (BDI-II)	Quantitative	748	14 to 19	64.7% females	Education	Fogarty International Centre, National Institute of Health; Ministry of Health, Jamaica	14.2% of participants reported depressive symptoms. There was an association between engagement in sexual activity, parental monitoring of adolescent activity, maternal affection and support, and paternal affection and support with self-reported depressive symptoms at the bivariate level.
Meeks Gardner, 2007	Jamaica	To examine risk factors for aggression among boys.	Behaviour and conduct problems	Author developed/adapted questionnaire	Quantitative	202	11.6 (0.6)	100% males	Education	UNICEF, Social Policy Analysis Project of the Planning Institute of Jamaica; Ministries of Health and of Education, Youth and Culture of Jamaica; Royal Netherlands Embassy	Exposure to neighbourhood violence, physical punishment at home and family discord were associated with increased risk; parents' being married, practising religion as a family and better school uniforms were associated with reduced risk.
Lipps, 2007	Jamaica	To explore the internal consistency reliability and the concurrent and discriminant validity of the Beck Depression Inventory II.	Depressive symptoms	Beck Depression Inventory–II (BDI-II); Centre for Epidemiology Studies-Depression (CES-D); Brief Screen for Depression (BSD); University of Los Angelos Loneliness Scale-Revised (UCLA-R)	Quantitative	690	23.4 (7.4)	77% females	Education	None reported	The BDI-II was found to have acceptable levels of reliability, concurrent and discriminatory validity.

Maharajh, 2006	Trinidad and Tobago	To investigate depression in adolescents and trends associated with its occurrence.	Depressive symptoms	Reynolds Adolescent Depression Scale (RADS)	Quantitative	1845	16.3 (1.13)	60% females	Education	None reported	14% of the sample was depressed. Depression was more likely to occur in females. The peak age was 16 years with a rate of 15.9%. Religion and prayer, intact family, and attendance at prestige schools was associated with lower depression rates. Adolescents with alcohol abuse in their family were more likely to be depressed.
Steely, 2006	Jamaica	To analyse relationships among perceived parental justness and harshness of corporal punishment, perceived parental acceptance-rejection, and psychological adjustment.	Multiple: psychological adjustment	Family History Assessment Form; Physical Punishment Questionnaire: Youth Form (Youth PPQ); Parental Acceptance-Rejection Questionnaire/Short Form (PARQ/Short Form); Personality Assessment Questionnaire: Child Version (Child PAQ)	Quantitative	97	7 to 18	55% females	Healthcare	None reported	Perceived harshness of punishment by itself had almost no direct effect on youths' psychological adjustment; however, the harsher the punishment was perceived to be, the more rejected youths felt. The more rejected youths felt, the more psychologically maladjusted they reported themselves to be.
Ramberan, 2006	Trinidad and Tobago	To investigate the correlates of body image perception.	Disordered Eating Behaviours and Attitudes	EATing Attitudes Test (EATS-26); Body Shape Questionnaire (BSQ-16); Body Silhouette Chart; Rosenberg Self-Esteem Scale (RSE); Drive for thinness subscale of the Eating Disorder Inventory 2.	Quantitative	251	16.3 (1.37)	100% females	Education	None reported	2.4% had a diagnosis of eating disorder. 51% had negative body image perception. Ethnicity was associated with concerns over body image, associated eating and weight-related behaviour.
Lipps, 2006	Jamaica	To explore the concurrent and discriminant validity of the Brief Screen for Depression.	Depressive symptoms	Brief Screen for Depression (BSD); Beck Depression Inventory (BDI-II); Centre for Epidemiology Studies-Depression (CES-D); University of Los Angelos Loneliness Scale-Revised (UCLA-R)	Quantitative	244	22.7 (not specified)	81.97% females	Education	None reported	The BSD was found to have acceptable concurrent and discriminant validity.
Marlowe, 2005	Bermuda	To study the interaction of ethnicity, culture, gender and economic factors that influence abnormal eating attitudes.	Disordered Eating Behaviours and Attitudes	Bulimic Investigatory Test, Edinburgh (BITE); Eating Attitudes Test (EAT-40)	Quantitative	836	12.2 (0.66)	52.5% females	Education	None reported	Lower socioeconomic status was associated with the risk of possible eating disorders. Developing anorexic eating attitudes were more prevalent compared to bulimic attitudes.
Maharajh, 2005	Trinidad and Tobago	To examine the epidemiology of suicidal behaviour and cannabis use among adolescents and explore relationships.	Suicidality	Community Assessment of Psychic Experiences (CAPE) questionnaire	Quantitative	227	12 to 20	Unclear	Education	None reported	Preliminary findings revealed that psychotic and depressive experiences were common in those using cannabis when compared to non-users. Cannabis use was higher in females at younger ages of 13 and 14.
Pottinger, 2005	Jamaica	To investigate children's experience of loss by parental migration.	Loss and grief	Author developed/adapted questionnaire	Quantitative	54	9 to 10	52% males	Education	None reported	Children's reactions were directly related to poor school performance and psychological difficulties. Being currently exposed to violence in the home and/or community was significantly associated with high scores on a measure of grief intensity. "Protective" factors included having someone to talk to

											about the migration and living in a supportive family.
Maharajh, 2005	Trinidad and Tobago	To investigate the occurrence of the most common mental disabilities.	Multiple	Case notes (DSM criteria)	Quantitative	349	14.4(2.85)	60% males	Healthcare	None reported	The most prominent diagnosis in younger children was learning disorders and ADHD. Conduct disorders, ADHD and depression were more common in children over 11.
Ali, 2004	Trinidad and Tobago	To examine suicidal behaviours through psychological autopsy, secondary data and psychiatric populations with an emphasis on causation and prevention.	Suicidality	The Suicidal Ideation Questionnaire (SIQ)	Quantitative	1845	14 to 20	60% females	Education	None reported	Gender differences existed for both suicidal ideation and attempts. Respondents from reconstituted families had higher suicidal ideation compared to other family structures, while intact families had the lowest rate for suicide attempts. Attendance to a religious institution lowered only suicidal ideation, while prayer with the family lowered both suicidal ideation and suicide attempts. Individuals with alcohol abuse in the family had higher suicidal ideation and attempts.
Maharajh, 2004	Trinidad and Tobago	To investigate the prevalence of depressive symptoms, major depression and dysthymia in Tobago.	Depressive symptoms	Reynolds Adolescent Depression Scale (RADS); Patient Health Questionnaires (PHQ-9)	Quantitative	198	16.3 (not specified)	60% females	Education	None reported	10.1% of adolescents experienced a depressive disorder, of which 6.06% was rated as dysthymia and 4.04 as major depression.
Halcón, 2003	Antigua, Barbados, Bahamas, BVI, Dominica, Grenada, Guyana, St. Lucia	To assess prevalence and related factors associated with youth health.	Multiple	Author developed/adapted questionnaire	Quantitative	15695	10 to 18	61% females	Education	Government of Italy; Pan American Health Organization ; University of Minnesota	Most adolescents saw themselves as generally happy. 1 in 6 were sad, angry, or irritable. 50% felt so “down” that they wondered if anything was worthwhile. Many of the young people reported a history of abuse.
Bhugra, 2003	Trinidad and Tobago, Barbados	To investigate eating attitudes and the prevalence of bulimic disorders.	Disordered Eating Behaviours and Attitudes	Bulimia Investigatory Test, Edinburgh (BITE); DSM-III-R Bulimia Diagnostic Interview	Quantitative	362	13 to 19	100% females	Education	None reported	0.8% scored over the cut-off point on the BITE. None of the interviewees was diagnosed as having bulimia nervosa. 67.7% reported being terrified of becoming fat and fat-fear was associated with higher Body Mass Index, dieting and exercising for losing weight.
Pottinger, 2003	Jamaica	To examine suicide attempts and ideation in children attending mental health clinics.	Suicidality	Case notes (DSM criteria)	Quantitative	57	12 (2.61)	60% females	Healthcare	None reported	Having poor relationships with primary carers and experiencing abuse were significantly associated with suicidal and externalising behaviours.
Naidu, 2002	Trinidad & Tobago	To investigate sources of stress and psychological disturbance in dental students.	Stress	Dental Environment Stress (DES)	Quantitative	94	22 (not specified)	55% females	Education	None reported	54.8 percent of males and 44.2 percent of females were in the clinical range indicating significant psychological disturbance. Psychological disturbance was significantly associated with stress levels for male students.
Baboolal, 2002	Trinidad and Tobago	To describe the profile of medical students accessing psychiatric services.	Multiple	Case notes (DSM IV criteria)	Quantitative	32	24.9 (2.83)	50% females	Education	None reported	Relationship issues were the most common stressor. Mood and anxiety disorders were present in 59% of students.
White, 2002	Jamaica	To investigate the occurrence of eating disorders.	Disordered Eating Behaviours and Attitudes	Author developed/adapted questionnaire	Quantitative	24	18.86 (not specified)	90.91% females	Healthcare	None reported	24 cases of Anorexia and Bulimia cases were identified. The authors concluded that the occurrence of eating disorders was very low.

Lambert, 2001	Jamaica	To investigate differences in behaviour and emotional problem ratings of teachers and observers.	Multiple: behavioural and emotional problems	Child Behaviour Checklist; Jamaican Teacher's Report Form	Mixed Methods	78	8.7 (1.59)	Unclear	Education	National Institute of Health; Michigan State University	Significantly higher problem scores emerged for ratings by observers than those by teachers. Observers also rated younger children as more demanding and aggressive while both informants rated rural children as exhibiting more externalizing problems than urban children.
Durbrow, 2000	St. Vincent and the Grenadines	To examine factors that undermine academic performance.	Multiple: anxiety and attention problems	Middle Childhood-Home Inventory; Learning Behaviours Scale; Revised Behaviour problem Checklist	Quantitative	61	5 to 14	69% males	Education	None reported	The findings suggested that academic performance can be improved in this population by reducing children's anxiety levels and promoting appropriate learning behaviours.
Foster-Williams, 2000	Jamaica	To assess psychological distress in younger siblings of patients with sickle cell.	Psychological distress	General Health Questionnaire (GHQ); Chronicity and General Health Questionnaire; Social Adjustment Scale - Modified	Quantitative	20	16.5 to 18	65% females	Healthcare	None reported	Although psychological distress appeared high, it did not significantly differ between patients, their sibling and controls.
Lambert, 1999	Jamaica	To assess behaviours of youth in the general public.	Behaviour and conduct problems	Jamaican Youth Checklist (JYC)	Quantitative	864	6 to 18	Unclear	Education	Institute for Public Policy and Social Research Excellence Award Fund; Michigan State University	Adolescents self-reported higher problem scores compared to the ratings they received from their parents and teachers. Girls received higher internalizing, and Somatic Complaints scores than boys. The clinical samples suggest that girls may not be receiving adequate services.
Perks, 1999	St. Lucia	To assess the effects of domestic violence on a non-clinical population of children and adolescents.	Depressive symptoms	Reynolds Adolescent Depression Scale (RADS); Reynolds Child Depression Scales (RCDS);	Quantitative	60	9 to 17	60% females	Education	None reported	Pupils whose parents were experiencing violent marital discord showed significantly higher levels of both depression and behavioural problems.
Phillips, 1997	Trinidad & Tobago	To determine the effectiveness/cultural appropriateness of the MMPI.	Personality	Minnesota Multiphasic Personality Inventory (MMPI)	Quantitative	448	15 to 20	54.91% females	Education	None reported	The findings suggest that with minor modifications the MMPI can be a useful instrument in the assessment of late adolescents in both the mental health and criminal justice systems in Trinidad and Tobago.
Hilton, 1997	Jamaica	To investigate the prevalence of psychiatric disorders.	Multiple	General health Questionnaire (GHQ); DSM; Modified social adjustment scale; Psychiatric assessment schedule	Quantitative	89	18 to 20	50.56% females	Healthcare	Wellcome Trust; Royal College of Psychiatrists	Rates of psychiatric disorders were 14% in males and 36% in females. Disorders were associated with female gender, unemployment, and social adjustment.
Deosaran, 1997	Trinidad and Tobago	To examine the social background and psychologicaldispositions of children in juvenile homes.	Behaviour and conduct problems	Author developed/adapted questionnaire	Quantitative	486	<18	75.31% males	Community Group Home	University of West Indies-CIDA Canadian Institutional Developmen t Agency	Most youths came fromworking class backgrounds and homes without two parents. The youths were predominantly of African descent (70%) and with only primary school education (over 50%). The youths expressed substantial optimism in improving themselves, and in accepting responsibility for their actions.
Foster-Williams, 1996	Jamaica	To examine the source of stress among medical students.	Stress	Questionnaire developed by the University of Texas.	Quantitative	180	University students	50.56% females	Education	None reported	The amount of content to be studied and exams and/or grades were rated as most stressful.

Lambert, 1994	Jamaica	To report empirical standardization of a child behaviour problem classification system.	Behaviour and conduct problems	Jamaican Teacher Check List (JTCL)	Quantitative	359	6 to 11	50.7% females	Education	University of Mississippi; Berger paints, Culture Originale Bridal Salon; Cremo Ltd; Eagle Merchant Bank; Facey Commodity; International Trust and Merchant Bank; Island Car Rental; Metal Box; Palomino Brothers; Sherwin Williams; Tropichem Laboratories ; Ultimate Services	The JTCL was found to be appropriate for use by clinicians and researchers.
Lambert, 1993	Jamaica	To compare teacher and parent ratings on child behaviour and emotional problems.	Behaviour and conduct problems	Jamaican Youth Checklist	Quantitative	359	6 to 11	50.42% females	Education	University of Mississippi; Berger paints, Culture Originale Bridal Salon; Cremo Ltd; Eagle Merchant Bank; Facey Commodity; International Trust and Merchant Bank; Island Car Rental; Metal Box; Palomino Brothers; Sherwin Williams; Tropichem Laboratories ; Ultimate Services	There was low to moderate correlations between parent and teacher reports on child behaviour and emotional problems.
Lambert, 1990	Jamaica	To examine teacher and parent reports of over and under controlled behaviours.	Multiple	Clinical interview (DSM criteria)	Quantitative	180	6 to 17	52.22% females	Healthcare	University of North Carolina	There were significant correlations between parent and teacher ratings of under controlled problems. The overcontrolled problem score between parents and teachers only significantly correlated for rural children and girls.

Richardson, 1989	Barbados	To inquire about the personality priorities of Caribbean adolescents.	Personality	Personality Priorities Inventory for Adolescents	Quantitative	194	15.6 (0.29)	58.76% females	Education	None reported	The rank order which emerged from the analysis indicated preference for Pleasing behaviours followed in order by Significance, Avoiding, and Detaching. Comparison between the sexes showed girls as being more inclined toward Pleasing, Significance, and Avoiding than boys.
Lambert, 1989	Jamaica	To identify over and under controlled behaviours.	Multiple	Clinician developed questionnaire (DSM-criteria)	Quantitative	320	6 to 17	50% females	Healthcare	University of North Carolina	Over and under controlled behaviours were identified. School avoidance was correlated with crying in children 6-11 and with adolescents 12-17.
Payne, 1988	Barbados	To identify the nature of adolescent fears.	Anxiety/Fear	Author developed/adapted questionnaire	Quantitative	657	12 to 15	53.27% females	Education	None reported	Fear of injury or death of self or loved ones, sexual concerns and fears of school failure were most common.
Payne, 1986	Barbados	To explore the attitudes of teachers, parents, and secondary school students towards behaviour problems.	Behaviour and conduct problems	Author developed/adapted questionnaire	Quantitative	297	14 to 15	82.5% females	Education	None reported	Parents rated most problems as more serious than teachers. Adults rated them higher than males, especially for conduct problems. Female students responded similarly to adults, except for heterosexual activity.
Burke, 1980	Jamaica	To investigate the aetiology of delinquency in the West Indies.	Behaviour and conduct problems	Not applicable	Quantitative	28	Mode 16 or 17	100% males	Healthcare	None reported	A third of a random sample of approved schoolboys had a neurotic disorder which can be treated by psychotherapy. Paternal deprivation and other factors were similarly distributed among personality disordered and neurotic delinquents in Jamaica.
Wray, 1976	Jamaica	To examine the effect of separation on clinical symptoms.	Multiple	Clinical interview (DSM criteria)	Quantitative	154	10 (3)	57.7% males	Healthcare	The Grant Foundation Inc	Separated children most frequently experienced emotional and conduct disorders.
